# Attitudes of the Lithuanian Population toward COVID-19 Vaccination and Evaluation of Its Effectiveness: A Cross-Sectional Study

**DOI:** 10.3390/medicina60020313

**Published:** 2024-02-12

**Authors:** Artur Airapetian, Benedikt Bachmetjev, Rolandas Zablockis

**Affiliations:** 1 Faculty of Medicine, Vilnius University, M.K. Ciurlionio 21, LT-03101 Vilnius, Lithuania; benedikt.bachmetjev@mf.stud.vu.lt; 2 Clinic of Chest Diseases, Immunology and Allergology, Institute of Clinical Medicine, Vilnius University, M.K. Ciurlionio 21, LT-03101 Vilnius, Lithuania; rolandas.zablockis@mf.vu.lt

**Keywords:** COVID-19, vaccination, attitude

## Abstract

*Background and Objectives:* This study delves into the attitudes, beliefs and determinants influencing the uptake of the COVID-19 vaccine among the Lithuanian population. *Materials and Methods:* Utilizing a cross-sectional study design, a total of 3166 respondents were surveyed. *Results:* The findings reveal a significant disparity in vaccination rates based on socio-demographic factors, with higher uptake observed among individuals with a university degree, urban residents and those in higher income brackets. Personal beliefs, particularly regarding the vaccine’s efficacy in pandemic management, played a pivotal role in vaccination decisions. This study also highlights the influence of external factors, such as the activity of the “anti-vaxxer” movement and the introduction of vaccination certificates. *Conclusions:* The results emphasize the need for targeted educational interventions and comprehensive public health campaigns to address vaccine hesitancy and promote widespread immunization.

## 1. Introduction

Cases of SARS-CoV-2 virus-induced atypical pneumonia were reported in Wuhan, China, in 2019. In February 2020, the World Health Organization declared it a global pandemic caused by the coronavirus disease [1]. COVID-19 is an infectious disease caused by severe acute respiratory syndrome coronavirus-2 (SARS-CoV-2). COVID-19 is most commonly transmitted by airborne droplets when an infected person coughs, sneezes or exhales, but it can also be transmitted by contact when a person touches his or her own eyes, nose or mouth after touching infected surfaces. The speed and severity of the spread of infection, rising morbidity and mortality rates and the impact of the demand for services on health systems require adequate infection management strategies and measures. The governments of several countries took aggressive measures to stop the virus from spreading all over the world. This procedure involved quarantine, isolation, mask wearing and social distancing. These measures have effectively slowed the spread of the epidemic, but severe acute respiratory syndrome coronavirus-2 (SARS-CoV-2) is still increasing worldwide [2].

In 2020, several COVID-19 vaccines were created and tested, and after regulatory authorization or approval for use and WHO recommendations [3,4], it was expected that efficacious vaccines would put an end to the COVID-19 pandemic [5]. Many countries began mass immunization programs for their citizens [6]. However, despite vaccines being available for society, individuals need to be encouraged to take the shot that prevents COVID-19 complications [7]. Even though these vaccinations are said to be safe and only occasionally result in minor side effects, allergy and thrombotic events have been linked to mortality [8]. Because of these unwelcome side effects and the lack of knowledge regarding the length of efficacy, many people were and are still skeptical of COVID-19 vaccinations [6]. However, taking into account the pandemic scenario and the benefit–risk ratio, the WHO authorized the use of vaccines for the wider population, especially for the most vulnerable part of society. Several countries have also begun mass immunization programs for their citizens [8].

In January 2021, Lithuania also started a mass inoculation program for the local population. However, most of the public was skeptical about immunization because of a lack of health literacy, political mistrust and the activity of the “anti-vaxxer” movement. The anti-vaccination movement’s initiators proved to be better persuaders because they used close sources of information—family members and people in the public eye—to communicate with people in a language they understood [9]. Nevertheless, vaccination coverage has increased significantly since the Ministry of Economy and Innovation announced the introduction of the vaccination certificate on 24 May 2021. Within 4 months, vaccination rates rose by 39%. This pandemic management measure caused a lot of controversy, as some people had to limit their social lives. However, citizens who wanted to lead a socially active life chose between the COVID-19 vaccine and the option of regular testing. This pandemic management strategy has attracted criticism and dissatisfaction in Lithuania, but statistics show that it has been an effective approach to pandemic management [10]. This led to the intention to investigate the reasons why the Lithuanian population chose the COVID-19 vaccination. This research reveals the real reasons why people in the country chose to vaccinate—whether it was a desire to protect themselves/others or whether it was a consequence of adapting to a political pandemic management tool. It is normal that vaccination, as a medical intervention, is not acceptable among the population. Even in a previous study, it was found that the COVID-19 vaccine acceptance rate in different countries varies. The acceptability percentage for vaccines is comparable in European nations, including Italy (53.7%), France (58.1%) and Poland (56.3%) [11]. Research has shown that 71.6% of the general population in Lithuania has been vaccinated for COVD-19. However, according to studies, a plethora of factors, including adverse health effects after vaccination, a lack of credible information and proper communication about safety and efficacy, long-term difficulties and a lack of trust in the current healthcare system, affect the attitudes of the general public toward COVID-19 vaccination [12]. The analysis showed that socio-demographic factors have an impact on how well-accepted the COVID-19 vaccine is. Therefore, this study was designed to identify public attitudes toward COVID-19 vaccination in terms of knowledge, attitude and perception. Accordingly, it is essential to start educating the public more intensively on vaccination issues by involving professionals from different fields, including doctors, scientists, journalists and politicians, in a more intensive discussion with the public on the most relevant science, health and technology topics, with the right messages to the public.

The impact of misinformation on public attitudes toward vaccination, particularly in the context of recent global health crises, is a critical area of concern. The spread of false information through various channels, notably social media, has emerged as a significant barrier to vaccine acceptance in many regions, including Europe and North America. This phenomenon underscores the urgent need for effective communication strategies to counteract misinformation and enhance public trust in health authorities and vaccines.

Misinformation, defined as false or misleading information presented as fact, has been particularly pervasive in the digital age, where social media platforms facilitate rapid information dissemination [13]. The ease with which misinformation spreads on these platforms can quickly lead to widespread public misconceptions about vaccination. For instance, false narratives around the safety and efficacy of vaccines have been shown to contribute to vaccine hesitancy, a reluctance or refusal to vaccinate despite the availability of vaccination services [14].

The consequences of misinformation are not merely theoretical but have tangible impacts on public health. In Europe and North America, where vaccine hesitancy fueled by misinformation has been notably prevalent, there has been a marked decline in vaccination rates, leading to the resurgence of diseases previously under control [15]. This decline underscores the critical need for effective communication strategies that can address and rectify misconceptions caused by misinformation.

Health communication strategies need to be multifaceted to be effective. They should not only provide accurate and transparent information about vaccines but also engage with the public to understand their concerns and build trust. The World Health Organization (WHO) emphasizes the importance of building trust in health authorities and the healthcare system as a cornerstone of increasing vaccine acceptance [16]. This involves proactive communication, transparency about vaccine development and safety and engagement with community leaders and influencers to disseminate accurate information.

Furthermore, studies have highlighted the role of tailored messaging and the use of behavioral insights in crafting communication strategies. For instance, some researchers suggest that messages that directly address specific misconceptions about vaccines can be more effective than generic pro-vaccine messages [17]. Additionally, leveraging social norms, such as highlighting the high percentage of people who vaccinate, can positively influence vaccination intentions [18].

This study represents a novel approach in examining the determinants of COVID-19 vaccine uptake, transcending traditional demographic factors such as education, age, income and urban residency. It uniquely investigates the sources from which individuals receive information regarding vaccine efficacy and composition. This aspect is crucial for health policymakers and AI specialists in devising targeted interventions across diverse media platforms, including social networks, television and radio. By evaluating the credibility of information disseminated on these platforms, this study aims to enhance strategies for promoting vaccination during pandemic crises, thereby contributing significantly to public health efforts [19].

## 2. Materials and Methods

### 2.1. Study Settings and Population

A cross-sectional study was conducted to identify the attitudes of the Lithuanian population toward COVID-19 vaccination. The target group was the general population in Lithuania. A convenient sampling technique was applied, and the study sample consisted of 3166 respondents.

### 2.2. Data Collection

When a respondent decided to participate in the study, informed consent was immediately obtained. After that, the questionnaire followed. In the informed consent process, participants received information regarding the study’s aims and objectives. Additionally, the participants were made aware that their data would be kept confidential. The questionnaire was addressed to the general public. It was created using the “Google” forms platform, and the sources were shared on the following social media sites: “Linkedin”, “Twitter” and “Facebook”. The Lithuanian population’s attitudes toward the COVID-19 vaccine were evaluated using questions about knowledge, attitudes and perceptions. The questionnaire consisted of five parts: respondents’ socio-demographic information, information on COVID-19 vaccination status, attitudes toward the effectiveness of vaccination for pandemic management, reasons for (not) taking the vaccine and where people obtain information about vaccination and its effectiveness.

### 2.3. Study Design

In this study, a cross-sectional study methodology was used. This survey received 3166 respondents, and there were no incorrectly filled-out questionnaires because it was online and respondents could not leave empty answers. Therefore, all responses were included in the study.

### 2.4. Inclusion and Exclusion Criteria

The study comprised members of the public who resided in Lithuania and had internet access. Participants were not approached personally or given anything in exchange for taking part in the study. In this study, there were no exclusions due to incorrectly filled-out surveys.

### 2.5. Statistical Analysis

Descriptive and analytical statistical methods were used to analyze the survey data. Prevalence estimates and 95% confidence intervals (CI) were calculated. Odds ratios (ORs) were estimated, and logistic regression models were constructed. Pearson’s χ^2^ test was used to detect differences in distributions across sociodemographic groups, and Fisher’s exact test was used to detect differences in the expected frequencies when the value per cell was less than 5. Mann–Whitney and Kruskal–Wallis tests were used to analyze the distributions of other dependent and independent variables. The differences were considered statistically significant when *p* ≤ 0.05. Data analysis was carried out using SPSS version 26.0 (IBM Corp, Armonk, NY, USA) and Rstudio version 4.2.2 (PBC Corp, Boston, MA, USA).

## 3. Results

### 3.1. Demographic Characteristics of the Participants

Table 1 displays the main characteristics of the respondents. Of the 3166 respondents that participated in the study, 22.5% were men and 77.5% were women. All of them were from different cities and suburbs. The majority of respondents were between the ages of 18 and 49 (76.7%). Equally, the majority of respondents had a university education (63.7%), which means an education higher than college. Almost one fifth of study participants had a college education (20.5%). The other groups in the academic classification were respondents with a high school diploma (14.1%) and those with a secondary school certificate (1.5%). The findings from the survey indicate that the income of participants’ households varied. Specifically, a minority of individuals reported earning less than 500 euros (13.5%). The majority of survey respondents were individuals whose per capita income fell within the EUR 500–1000 (39.0%) and EUR 1000–2000 (33.5%) brackets. Therefore, the respondents who made more than EUR 2000 per person per month in their households made up a minor percentage of the total (13.9%). Most of the participants (81.6%) indicated that they lived in urban areas, with the rest (18.4%) living in rural areas.

### 3.2. Main Study Results

In the survey, 71.6% of study participants reported that they had been vaccinated for COVID-19. Of the survey’s male participants, 69.0% had a vaccination, and 72.4% of the study’s female participants had COVID-19 jabs (Table 2). Within 71.6% of vaccinated respondents, only 21.7% were men due to the low male response rate, and 78.3% were women. Of respondents, 61.6% agreed that the COVID-19 vaccine is an effective tool for pandemic management. Of male and female respondents, 60.2% and 62.0%, respectively, equal to 61.6% of all respondents, agreed that the COVID-19 vaccine is an effective tool for pandemic management. In order to clarify the effect modifiers, people were asked, “Have you been vaccinated with vaccines included in the vaccination calendar?” For this, 70.8% of respondents answered ‘Yes’, of whom 62.4% were men and 73.2% were women. Moreover, 5.5% of people reported their earlier immunizations, of which 8.1% were male and 4.8% were female. In addition, 15.5% of people surveyed, of whom 17.3% were male and 15% were female, confirmed that they “have been immunized but not with all vaccines that are part of the vaccine calendar”. Of men and women, 8.1% and 4.8%, respectively, were among the 8.2% who were unsure if they had been immunized with a jab on the vaccination schedule. When asked about “what should be the most appropriate COVID-19 vaccination model in Lithuania”, the majority of respondents (60.0%) said that the vaccination process should be a free choice. A third of respondents (29.7%) expressed the opinion that vaccination requirements for specific activities should be mandatory, i.e., visiting stores, entertainment, etc. Of the population, 10.3% believe that mandatory vaccinations for all individuals should be implemented (Table 2).

#### 3.2.1. Reasons to Refuse or Receive the COVID-19 Vaccine

**Figure 1:**
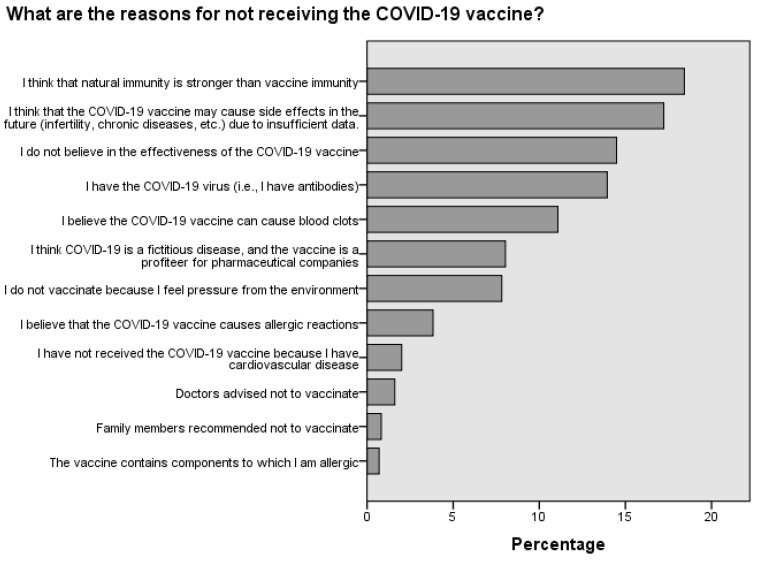
Reasons for vaccination against COVID-19.

**Figure 2:**
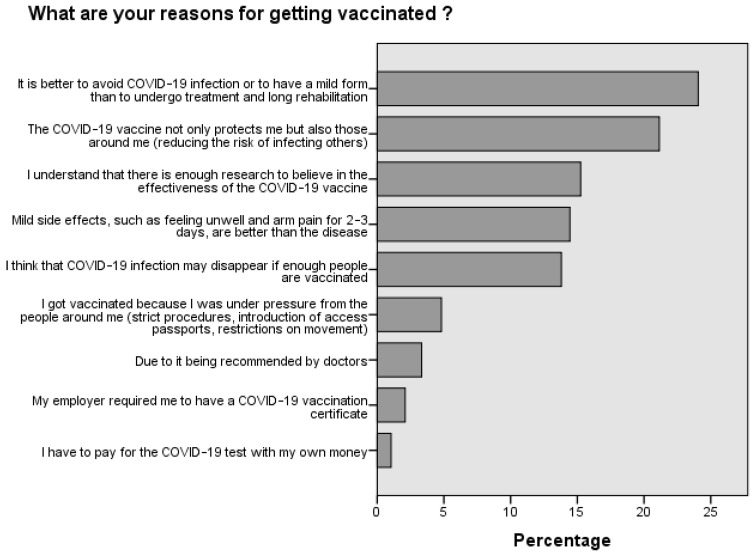
Reasons for not vaccinating against COVID-19.

The main reason (Figure 1) for vaccinating against COVID-19 was preventive motivation, meaning the belief that it is better to avoid the disease than to have to undergo treatment and a long rehabilitation period, accounting for 24.1% of respondents. Moreover, 21.1% of respondents were vaccinated because of their responsibility for others. The most common reasons for not vaccinating (Figure 2) were as follows: 18.4% of respondents trusted natural immunity; 17.2% decided not to vaccinate due to a lack of information on vaccine side effects; and 14.4% of respondents expressed a lack of confidence in the effectiveness of vaccines.

#### 3.2.2. Where Do People Seek Details about the Chemical Composition and Efficacy of Vaccines?

**Figure 3:**
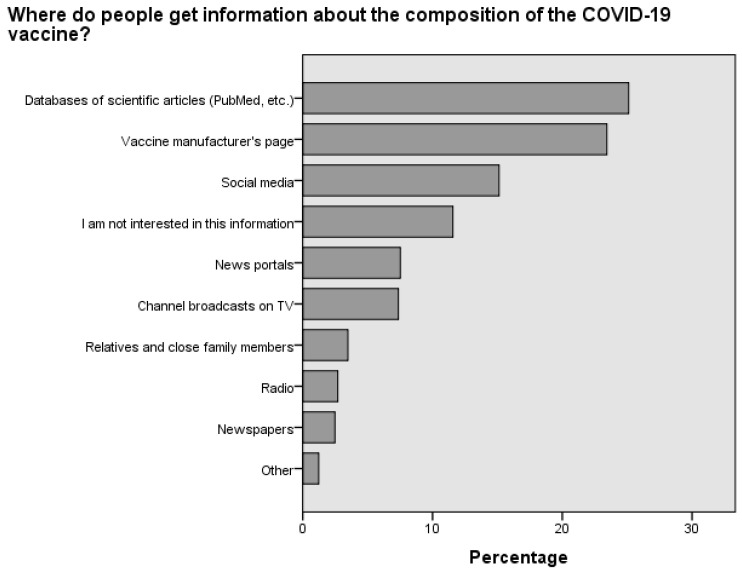
Distribution of preferred information sources regarding the composition of the COVID-19 vaccine among Lithuanian respondents.

**Figure 4:**
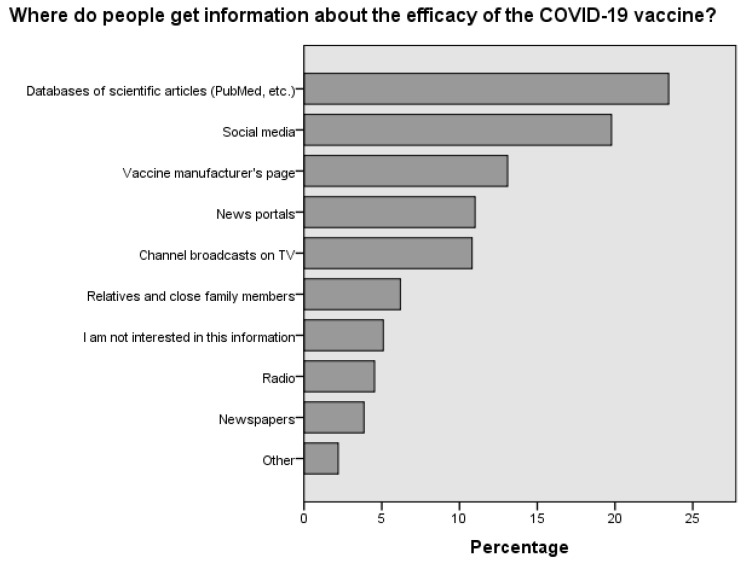
Distribution of preferred information sources regarding the efficacy of the COVID-19 vaccine among Lithuanian respondents.

The data indicate that the majority of individuals sought information about the vaccine’s composition in scientific articles (25.1%) and on the manufacturer’s website (23.4%). Additionally, a considerable number of people searched for details about the vaccine’s composition on social networks (15.1%) (Figure 3). The survey of respondents showed a similar trend regarding searching for information about the vaccine’s effectiveness. Most respondents relied on scientific publications (23.5%), social networks (19.8%) and the manufacturer’s website (13.1%) to obtain this information (Figure 4).

**Figure 5:**
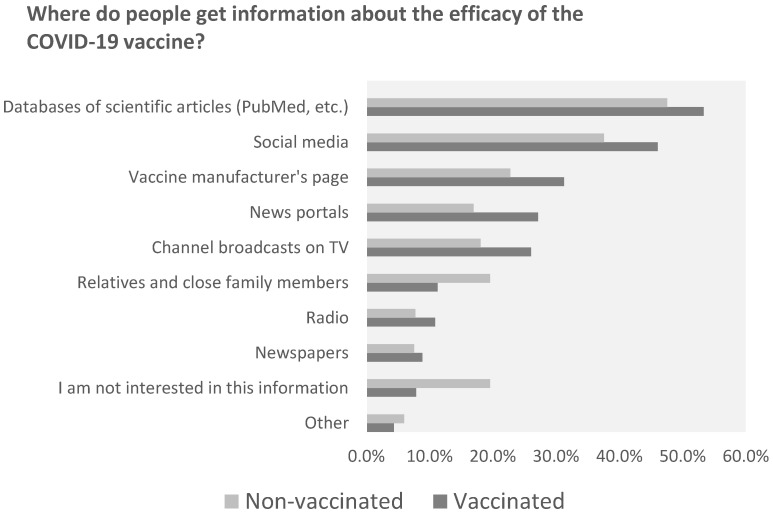
Vaccinated and unvaccinated groups’ use of information sources.

Upon assessing the sources from which individuals gather information about COVID-19 vaccine efficacy, clear distinctions emerge between the vaccinated and non-vaccinated populations. Scientific databases like PubMed were the most favored resource for the vaccinated group, with over half (53.4%) consulting these for information, indicating strong trust in academic research. The non-vaccinated also utilized these databases but to a lesser degree (47.6%). Social media platforms were significantly more influential among the vaccinated (46.1%) than the non-vaccinated (37.6%), suggesting a substantial role for networks like Facebook and Twitter in vaccination decisions. Conversely, non-vaccinated individuals showed a higher tendency (19.6%) to rely on information from their families and close relatives, compared to 11.3% of the vaccinated group, highlighting the impact of personal connections on vaccine skepticism. Professional news outlets were frequented more by the vaccinated (27.2%) than the non-vaccinated (17%), pointing toward greater trust in journalistic sources for vaccine information among the former. Strikingly, there was a notable lack of interest in vaccine efficacy information among non-vaccinated individuals (19.6%), which was more than double the rate seen in the vaccinated group (7.9%), which could be a factor in vaccine hesitancy. Television and radio had a broader audience within the vaccinated group (TV: 26.1%, radio: 10.9%) as opposed to the non-vaccinated group (TV: 18.1%, radio: 7.8%), indicating traditional media’s continued influence on those who choose to vaccinate. To summarize, the vaccinated were more inclined toward scientific, social media and news outlets for vaccine information, whereas the non-vaccinated were influenced more by personal networks and demonstrated a significant disinterest in vaccine-related information, which is critical to understanding the dynamics of vaccine acceptance and hesitancy. Traditional media also played a significant role in reaching the vaccinated populace (Figure 5).

### 3.3. Vaccination Coverage

Starting with age, we observed a trend: younger individuals, specifically those between 18 and 29, were the most proactive, with 76.1% getting vaccinated. As age increased, there was a slight dip in these numbers, with 70.4% in the 30–39 bracket and 69.9% in the 40–49 bracket getting the jab. Interestingly, those above 50 years showed a resurgence in interest, with a 71.4% vaccination rate (Table 3). The urban–rural divide was evident in our findings. City residents were more inclined to get vaccinated, with a rate of 73.0%, compared to their rural counterparts, who had a 65.3% vaccination rate (Table 3). Education emerged as another influential factor. University graduates were more likely to be vaccinated at 77.1%, compared to those without such a degree, where the rate stood at 61.9%. (Table 3) Income, too, played a role. Those earning up to EUR 500 had a 63.1% vaccination rate. This percentage saw a steady rise with increasing income brackets, peaking at 74.7% for those earning between EUR 1000 and 2000, before slightly dropping to 69.5% for individuals with incomes above EUR 2000 (Table 3). This was the biggest difference, but it is not unexpected or striking that those convinced of the vaccine’s role in managing the pandemic opted for it, whereas, among the skeptics, only 31.0% chose to get vaccinated (Table 3). Past behaviors also influenced current decisions. Those who had been consistent with their previous vaccinations were more likely to get the COVID-19 vaccine, with a rate of 74.0%. In contrast, only 50.3% of those who had not adhered to past vaccination schedules got the COVID-19 shot (Table 3). Last, opinions on vaccination policies in Lithuania provided insightful data. A significant majority of 99.4% who advocated for mandatory vaccination received the vaccine. Similarly, 99.3% of those who felt vaccination should be tied to certain activities, like shopping, got vaccinated. However, among those favoring optional vaccination, the rate was considerably lower at 53.2% (Table 3).

In terms of age distribution, belief in the vaccine’s efficacy was relatively consistent. For instance, among those aged 18–29, 63.3% found it effective, and this sentiment was echoed in other age groups, with the over-50 group having a slightly lower rate at 59.3%. This suggests that age did not significantly influence opinions on the vaccine’s effectiveness (Table 4). When considering the place of residence, urban dwellers were more convinced of the vaccine’s role, with 63.9% endorsing its effectiveness, compared to 51.0% in rural areas. This indicates that urban and rural environments might shape vaccine attitudes differently (Table 4). Education also played a pivotal role in shaping perceptions. University graduates were more positive about the vaccine, with 70.2% in favor, as opposed to 46.3% among those without a degree. This suggests that higher education might be linked to a more favorable view of the vaccine (Table 4). Income level further influenced perceptions. Those in the lowest income bracket (below EUR 500) were the most skeptical, with 47.2% affirming the vaccine’s role. This trust grew with income, suggesting that financial standing might be tied to vaccine perceptions (Table 4). Interestingly, those who had already received the COVID-19 vaccine were overwhelmingly positive about its effectiveness, with 83.4% in favor. In stark contrast, only 6.6% of the unvaccinated group shared this sentiment. This stark difference underscores the role that personal experience with the vaccine might play in shaping views (Table 4). Furthermore, this study found that 65.3% of respondents who had received vaccinations from the standard vaccination calendar believed in the COVID-19 vaccine’s effectiveness. In contrast, only 29.1% of those who had not been immunized with standard vaccinations shared this belief. This indicates that past vaccination behaviors might influence current perceptions (Table 4). Last, opinions on the vaccination model in Lithuania also influenced perceptions. For example, 63.5% of those who believed vaccinations should be a matter of personal choice found the COVID-19 vaccine effective. In contrast, nearly all of those who believed in mandatory vaccinations or tying vaccinations to specific activities like shopping overwhelmingly believed in the vaccine’s effectiveness (Table 4).

### 3.4. Factors Affecting Vaccination: Logistic Regression

A binominal regression model was developed to predict the vaccination rate for different socio-demographic variables. The dependent variable was the following question: “Have you been vaccinated with the COVID-19 vaccine?” The independent variables were age, place of residence, level of education and per capita income (Table 5).

Another logistic regression model was developed to investigate the relationship between the COVID-19 vaccine and people’s opinions regarding its effectiveness as a tool for pandemic management, vaccination status with respect to vaccines included in the vaccination calendar and preferences for the vaccination model used in Lithuania. This model aims to provide insight into the factors influencing vaccination rates in the population (Table 6.)

### 3.5. Factors Affecting Opinions Related to COVID-19 Vaccine Efficacy: Logistic Regression

In this study, a logistic regression model was developed to examine the relationship between people’s perceptions of the effectiveness of the COVID-19 vaccine and various socio-demographic, opinion and attitude factors. The primary aim of this model was to gain a deeper understanding of the factors that influence the public’s perceptions of the vaccine’s efficacy in managing the ongoing pandemic. The model takes into account various socio-demographic factors such as age, education level and monthly capital, as well as opinion and attitude factors such as doubts about vaccines that are on the vaccination calendar and dissatisfaction with the vaccination pattern in the country. By including these variables in the model, this study aimed to identify the most important factors influencing public attitudes toward vaccine effectiveness. Notably, the model was not partitioned into separate parts to distinguish between socio-demographic and opinion factors due to concerns about the overall goodness of fit of the model. This decision was made in recognition of the complex and multifaceted nature of the factors under investigation and the potential for significant interplay between these variables (Table 7).

## 4. Discussion

The present study provides comprehensive insight into the attitudes, beliefs and factors influencing the uptake of the COVID-19 vaccine among the Lithuanian population. The findings of this study are consistent with global trends, in which vaccine hesitancy is influenced by a myriad of factors ranging from socio-demographic characteristics to personal beliefs and external influences [14].

A significant observation from this study is the disparity in vaccination rates based on socio-demographic factors. The higher uptake of the vaccine among individuals with a university degree, those residing in urban areas and those with a higher income is consistent with findings from other studies [20]. This could be attributed to better access to information, higher health literacy and greater trust in the healthcare system among these groups [21]. The role of education in influencing vaccine uptake cannot be understated. As observed, individuals with higher education levels were more likely to perceive the vaccine as an effective tool against the pandemic. This underscores the importance of targeted educational interventions to address vaccine hesitancy among those with lower educational attainment [22].

The influence of personal beliefs and attitudes on vaccine uptake was evident in this study. The perception of the vaccine’s effectiveness played a pivotal role in determining whether an individual chose to get vaccinated. This is in line with the Health Belief Model, which posits that individuals are more likely to take preventive health actions if they perceive a threat and believe that the action will mitigate the threat [23]. The fact that a significant proportion of the population believed in the efficacy of natural immunity over vaccine-induced immunity highlights the need for public health campaigns to address misconceptions and provide evidence-based information on the benefits of vaccination [24].

The role of external influences, particularly the “anti-vaxxer” movement, was evident in this study. The ability of the anti-vaccination movement to leverage close sources of information and communicate in a language that resonates with the public underscores the challenges faced by public health authorities in promoting vaccination [25]. The introduction of the vaccination certificate and its subsequent impact on vaccination rates is a testament to the power of policy interventions in influencing public behavior. However, it also raises ethical questions about the balance between individual rights and public health imperatives [26].

The sources of information that individuals relied upon for vaccine-related information were diverse. The reliance on scientific articles and manufacturer websites is encouraging, as it indicates a preference for evidence-based information. However, the significant proportion of individuals who sought information from social networks is cause for concern given the potential for misinformation on these platforms [27].

Comparing vaccination attitudes and behaviors in Poland with those of its neighboring country, Lithuania, reveals notable differences. In Lithuania, a majority of the younger population appears more inclined toward vaccination compared to its counterpart in Poland. For both countries, individuals over the age of 50 exhibit high vaccination rates, representing the most active demographic in this regard [28].

In Poland, there is a discernible gender disparity in the perception of vaccination effectiveness, with men generally perceiving vaccines as more reliable than how women perceive them. Conversely, in Lithuania, the assessment of vaccine effectiveness is relatively uniform across different genders. A particularly interesting observation in Poland is the positive correlation between age and confidence in vaccine effectiveness; this trend is reversed in Lithuania, where older individuals, despite being more likely to get vaccinated, exhibit the highest levels of vaccine non-confidence [28].

The urban–rural divide also presents contrasting patterns in these countries. In Poland, the perception of vaccine effectiveness is fairly consistent between urban and rural areas. However, in Lithuania, a significant disparity exists, with urban residents demonstrating greater confidence in vaccine effectiveness compared to their rural counterparts [28].

This study, conducted in Lithuania, is notably more robust, involving a larger sample size of 3166 respondents. Beyond mere frequency calculations, it employs logistic regression models to predict vaccination status. For instance, individuals with university education are 2.12 times (odds ratio [OR] of 2.12, Table 5) more likely to be vaccinated compared to those without higher education. Similarly, urban residents are 1.22 times (OR of 1.22, Table 5) more likely to be vaccinated than rural inhabitants. This study also indicates that individuals over 50 years old are 0.72 times (OR 0.72, Table 5) more likely to be vaccinated than those aged 18–29.

Income levels also correlate with vaccination likelihood; individuals earning between EUR 500 and 1000 per month are 1.3 times (OR 1.3, Table 5) more likely to be vaccinated than those earning below EUR 500. This trend persists for individuals earning between EUR 1000 and 2000 per month. Furthermore, belief in vaccine effectiveness dramatically increases the likelihood of vaccination, with a 28.45 times (OR of 28.45, Table 6) higher probability among believers. Similarly, those advocating for mandatory vaccination are 16.68 times (OR of 16.68, Table 6) more likely to be vaccinated. The predictive model extends these findings, estimating group-specific attitudes toward vaccine effectiveness (Table 7).

Considering the varying vaccination rates across different countries, age groups and cities, this study distinguishes itself by examining the sources from which individuals gather information about vaccine composition (Figure 3) and effectiveness (Figure 4) [28,29,30,31]. A notable finding is the significant influence of not only social media but also scientific journals that discuss common complications. These complications include cerebrovascular disorders, such as cerebral venous sinus thrombosis, transient ischemic attack, intracerebral hemorrhage, ischemic stroke and demyelinating disorders like transverse myelitis, first manifestations of multiple sclerosis (MS) and neuromyelitis optica [32,33]. This study’s results indicate that approximately 47.6% of individuals who chose not to vaccinate (Figure 5) regularly read scientific journals. This study is of paramount importance, as it highlights the necessity for concerted efforts by medical professionals, public health authorities and policymakers to communicate that, although such complications can occur, they are rare. By doing so, we can alleviate fears and enhance vaccination rates, not only in Lithuania, which is centrally located in Europe, but also globally.

## 5. Conclusions

Vaccination uptake was influenced by higher education, a higher income level, place of residence, favorable attitudes toward the effectiveness of the COVID-19 vaccine in pandemic management and support for the mandatory COVID-19 vaccination model. Attitudes toward the vaccine’s effectiveness were influenced by higher education, a more mature age, a higher income level, support for the mandatory COVID-19 vaccination model and vaccination with vaccines that are part of the vaccination calendar. Most people received the COVID-19 vaccine because they wanted to protect themselves from the disease and avoid potentially serious complications that would require lengthy treatment and rehabilitation. Most of those who were not vaccinated said that natural immunity is stronger than immunity acquired by the vaccine. It should be noted that a significant proportion of individuals consulted scientific publications regarding the efficacy of the vaccine, inclusive of those who had not received vaccination. However, it is concerning that approximately one-fifth of respondents sought information from social networks and the websites of vaccine manufacturers. Additionally, a tenth of respondents relied on information from news portals and television programs.

## 6. Recommendation

The World Health Organization classified vaccine hesitancy as one of the biggest threats to world health even before the current outbreak [34]. In this study, we found that the groups of people least likely to be vaccinated are older people, those living in rural areas and those without higher education. Most of those who had not been vaccinated did not believe in the effectiveness of vaccination and thought that it could cause many side effects. This article highlights a target group of people that could be better educated, and the availability of information on the benefits of the COVID-19 vaccine could be increased. In this way, we will be able to solve one of the world’s prevailing problems by educating the target group of people that this study aimed to discover.

## Figures and Tables

**Table 1:** Demographic characteristics of the respondents.

Demographic Variable		Number of Participants (Percentage)
Gender	Male	713 (22.5)
	Female	2453 (77.5)
Age	18–29	591 (18.7)
	30–39	1005 (31.7)
	40–49	831 (26.3)
	>50	739 (23.3)
Education	University education	2018 (63.7)
	Non-university education	1148 (36.3)
Per capita household income (euros)	Less than 500	426 (13.5)
	500–1000	1237 (39.1)
	1000–2000	1063 (33.6)
	>2000	440 (13.9)
Place of residence	Urban	2584 (81.6)
	Rural	582 (18.4)

**Table 2:** Association between COVID-19 vaccination status, attitudes toward vaccination and socio-demographic variables.

Questions			Gender	Participant Responses			
				Yes		No	
Have you been vaccinated against the COVID-19 infection?			Male	492 (69.0)		221 (31.0)	
			Female	1775 (72.4)		678 (27.6)	
Is the COVID-19 vaccine an effective tool for pandemic management?			Male	429 (60.2)		284 (39.8)	
			Female	1520 (62.0)		933 (38.0)	
				Participant Responses			
				Positive	Negative	Unknown	Incomplete *
Vaccination calendar status **			Male	445 (62.4)	58 (8.1)	87 (12.2)	123 (17.3)
			Female	1796 (73.2)	117 (4.8)	172 (7.0)	368 (15.0)
				Participant Responses			
				No		Partially ***	Yes
Should COVID-19 vaccination be mandatory?			Male	402 (56.4)		215 (30.2)	215 (13.5)
			Female	1500 (61.1)		724 (29.5)	229 (9.3)

* I have been immunized but not with all the vaccines included in the vaccine calendar; ** Vaccination status according to the immunization schedule; *** Vaccination should be mandatory for specific activities. Data are shown as numbers (%).

**Table 3:** Associations between socio-demographic factors, attitudinal dispositions and COVID-19 vaccination status among Lithuanian respondents.

		COVID-19 Vaccination Status		*p*-Value
		Positive	Negative	
Gender	Male	492 (69.0)	221 (31.0)	0.080
	Female	1775 (72.4)	678 (27.6)	
Age (years)—mean ± SD		40.4 ± 12.2	41.0 ± 11.4	0.197
Age	18–29	450 (76.1)	141 (23.9)	**0.049**
	30–39	708 (70.4)	297 (29.6)	
	40–49	581 (69.9)	250 (30.1)	
	>50	528 (71.4)	211 (28.6)	
Place of residence	Urban area	1887 (73.0)	697 (27.0)	**<0.001**
	Rural area	380 (65.3)	202 (34.7)	
Education	University	1556 (77.1)	462 (22.9)	**<0.001**
	Non-university	711 (61.9)	437 (38.1)	
Monthly income per person in the household (EUR)	<500	269 (63.1)	157 (36.9)	**<0.001**
	500–1000	898 (72.6)	339 (27.4)	
	1000–2000	794 (74.7)	269 (25.3)	
	>2000	306 (69.5)	134 (30.5)	
Is the COVID-19 vaccine an effective tool for pandemic management?	Yes	1890 (97.0)	59 (3.0)	**<0.001**
	No	377 (31.0)	840 (69.0)	
Vaccination calendar status **	Positive	1659 (74.0)	582 (26.0)	**<0.001**
	Negative	88 (50.3)	87 (49.7)	
	Unknown	197 (76.1)	62 (23.9)	
	Incomplete *	323 (65.8)	168 (34.2)	
Should COVID-19 vaccination be mandatory?	No	1012 (53.2)	890 (46.8)	**<0.001**
	Partially ***	932 (99.3)	7 (0.7)	
	Yes	323 (99.4)	2 (0.6)	

* I have been immunized but not with all the vaccines included in the vaccine calendar; ** Vaccination status according to immunization schedule; *** Vaccination should be mandatory for specific activities. Data are shown as the mean ± SD or as numbers (%). Significant values are shown in bold.

**Table 4:** Relationship between perceived efficacy of the COVID-19 vaccine and socio-demographic and vaccination attitude variables.

		Is the COVID-19 Vaccine an Effective Tool for Pandemic Management?		*p*-Value
		Yes	No	
Gender	Male	429 (60.2)	284 (39.8)	0.385
	Female	1520 (62.0)	933 (38.0)	
Age (years)—mean ± SD		40.3 ± 11.9	40.9 ± 12.0	0.182
Age	18–29	374 (63.3)	217 (36.7)	0.431
	30–39	617 (61.4)	388 (38.6)	
	40–49	520 (62.6)	311 (37.4)	
	>50	438 (59.3)	301 (40.7)	
Place of residence	Urban area	1652 (63.9)	932 (36.1)	**<0.001**
	Rural area	297 (51.0)	285 (49.0)	
Education	University	1417 (70.2)	601 (29.8)	**<0.001**
	Non-university	532 (46.3)	616 (53.7)	
Monthly income per person in the household (EUR)	<500	201 (47.2)	225 (47.2)	**<0.001**
	500–1000	744 (60.1)	493 (39.9)	
	1000–2000	719 (67.6)	344 (32.4)	
	>2000	285 (64.8)	155 (35.2)	
COVID-19 vaccination status	Yes	1890 (83.4)	377 (16.6)	**<0.001**
	No	59 (6.6)	840 (93.4)	
Vaccination calendar status **	Positive	1463 (65.3)	778 (34.7)	**<0.001**
	Negative	51 (29.1)	124 (70.9)	
	Unknown	172 (66.4)	87 (33.6)	
	Incomplete *	263 (53.6)	228 (46.4)	
Should COVID-19 vaccination be mandatory?	No	694 (36.5)	1208 (63.5)	**<0.001**
	Partially ***	934 (99.5)	5 (0.5)	
	Yes	321 (98.8)	4 (1.2)	

* I have been immunized but not with all the vaccines included in the vaccine calendar; ** Vaccination status according to immunization schedule; *** Vaccination should be mandatory for specific activities. Data are shown as the mean ± SD or as numbers (%). Significant values are shown in bold.

**Table 5:** Binominal logistic regression model that predicts vaccination status according to sociodemographic variables.

Independent Variable	Reg. Coefficient β	Std. Error	Std. Deviation	Odds Ratio (95% CI)	*p*-Value
Constant term	0.476	0.151	3.154	-	0.002
Age group:					
30–39 years	−0.465	0.123	−3.775	0.63 (0.49–0.80)	**<0.001**
40–49 years	−0.479	0.126	−3.786	0.62 (0.48–0.79)	**<0.001**
>50 years	−0.330	0.130	−2.538	0.72 (0.56–0.93)	**0.011**
Place of residence	0.198	0.102	1.944	1.22 (1.00–1.49)	0.052
Level of education	0.751	0.086	8.745	2.12 (1.79–2.51)	**<0.001**
Income per capita:					
EUR 500–1000/month	0.269	0.123	2.187	1.30 (1.03–1.66)	**0.030**
EUR 1000–2000/month	0.273	0.129	2.108	1.31 (1.02–1.69)	**0.035**
>EUR 2000/month	−0.006	0.153	−0.038	0.99 (0.74–1.34)	0.970

Significant values are shown in bold.

**Table 6:** Binominal logistic regression model that predicts vaccination status according to a respondent’s opinions and attitudes toward vaccines.

Independent Variable	Reg. Coefficient β	Std. Error	Std. Deviation	Odds Ratio (95% CI)	*p*-Value
**Constant term**	−0.806	0.189	−4.267	-	**<0.001**
**Vaccine efficiency opinion**	3.348	0.157	21.330	28.45 (21.10–39.07)	**<0.001**
**Vaccination status by vaccination calendar**					
Positive	−0.023	0.202	−0.113	0.98 (0.66–1.46)	0.910
Negative	−0.044	0.232	−0.191	0.96 (0.61–1.51)	0.910
Unknown	0.118	0.280	0.423	1.13 (0.65–1.95)	0.672
**Should COVID−19 vaccination be mandatory?**					
Partially *	2.480	0.404	6.138	11.94 (5.79–28.91)	**<0.001**
Yes	2.814	0.727	3.870	16.68 (5.09–102.78)	**<0.001**

* Vaccination should be mandatory for specific activities. Significant values are shown in bold.

**Table 7:** Binominal logistic regression model that predicts a prospective respondent’s attitudes toward vaccines depending on different circumstances.

Independent Variable	Reg. Coefficient β	Std. Error	Std. Deviation	Odds Ratio (95% CI)	*p*-Value
**Constant term**	−4.887	0.368	−13.271	-	<0.001
**Age group:**					
30–39 years	0.240	0.184	1.305	1.27 (0.89–1.82)	0.192
40–49 years	0.380	0.193	1.965	1.46 (1.00–2.14)	**0.049**
>50 years	0.211	0.193	1.096	1.24 (0.85–1.80)	0.273
**Place of residence**	0.164	0.154	1.068	1.18 (0.87–1.59)	0.285
**Level of education**	0.902	0.133	6.798	2.46 (1.90–3.20)	**<0.001**
**Income per capita:**					
EUR 500–1000/month	0.113	0.187	0.604	1.12 (0.78–1.62)	0.546
EUR 1000–2000/month	0.400	0.200	2.002	1.49 (1.00–2.21)	0.045 *
>EUR 2000/month	0.341	0.249	1.373	1.41 (0.87–2.29)	0.170
COVID-19 vaccination status	3.401	0.161	21.137	29.98 (22.06–41.49)	**<0.001**
Vaccination calendar status **					
Positive	1.035	0.274	3.783	2.82 (1.66–4.88)	**<0.001**
Incomplete *	0.717	0.302	2.374	2.05 (1.14–3.75)	**0.018**
Unknown	1.122	0.336	3.341	3.07 (1.60–5.99)	**<0.001**
**Should COVID-19 vaccination be mandatory?**					
Partially ***	4.809	0.459	10.481	122.59 (55.42–348.08)	**<0.001**
Yes	4.015	0.524	7.657	55.44 (22.58–185.47)	**<0.001**

* I have been immunized but not with all the vaccines included in the vaccine calendar; ** Vaccination status according to immunization schedule; *** Vaccination should be mandatory for specific activities. Significant values are shown in bold.

## Data Availability

The data that support the findings of the study are available from the corresponding author upon reasonable request.
